# Association between childhood obesity and infertility in later life: a systematic review of cohort studies

**DOI:** 10.1186/s12902-023-01490-4

**Published:** 2023-10-24

**Authors:** Farzad Pourghazi, Maysa Eslami, Sammy Mohammadi, Reza Ghoreshi, Hanieh-Sadat Ejtahed, Mostafa Qorbani

**Affiliations:** 1 Endocrinology and Metabolism Research Center, Endocrinology and Metabolism Clinical Sciences Institute, Tehran University of Medical Sciences, Tehran, Iran; 2 Non-communicable Diseases Research Center, Alborz University of Medical Sciences, Karaj, Iran; 3 Obesity and Eating Habits Research Center, Endocrinology and Metabolism Clinical Sciences Institute, Tehran University of Medical Sciences, Tehran, Iran; 4 Chronic Diseases Research Center, Endocrinology and Metabolism Population Sciences Institute, Tehran University of Medical Sciences, Tehran, Iran

**Keywords:** Childhood obesity, Fertility, Infertility, Systematic review

## Abstract

**Background:**

The global prevalence of childhood obesity has exhibited a troubling surge in recent years. Due to the raised questions regarding its potential correlation with infertility in adulthood, this systematic review has been undertaken to explore the relationships between childhood obesity, and infertility later in life.

**Methods:**

A comprehensive search was performed in three international databases (PubMed, Web of Science, and Scopus). All cohort (retrospective or prospective), case-cohort, and nested case-control studies until April 2022 which assessed the association of obesity in children and adolescents with male and female infertility indicators in later life were included. The quality of the included studies was assessed by Newcastle-Ottawa quality assessment checklists.

**Result:**

Out of the initial 32,501 documents, eleven eligible studies with a total sample size of 498,980 participants were included. Five studies focused on the number of offspring and indicated that obesity, especially in adolescence had an association with later life lower number of children, nulliparity, and childlessness in both men and women. Concerning conceiving problems, two studies showed that obesity before age 12 increased the risk of female fertility problems in the future. Two studies reported that obesity in early life raised the risk of impaired female reproductive system such as menstrual or ovulatory problems. As well as females, a study discovered that obesity in men during their 20s was linked to an elevated risk of low sperm motility and poor sperm morphology. Another study has reported men with higher pre-pubertal BMI had lower sex hormone-binding globulin; however, the same association was not seen between childhood BMI and semen quality.

**Conclusion:**

The evidence suggests a positive association between childhood obesity with infertility indicators in later life. Childhood weight reduction strategies are suggested to be implemented in societies in order to reduce infertility rates in later life.

**Supplementary Information:**

The online version contains supplementary material available at 10.1186/s12902-023-01490-4.

## Introduction

Obesity and overweight in early life remain a global concern in recent years because the prevalence of childhood and adolescence obesity holds consequences for various dimensions of an individual’s overall health [1–5]. The prevalence of childhood obesity has exhibited a persistent upward trajectory in recent years. Consequently, the concern among healthcare professionals and governmental bodies has intensified significantly [6]. throughout the past decades, he health and fitness status observed in early adulthood has experienced a decline, even as the incidence of obesity and overweight has increased. This rise can be attributed to lower rates of physical activity among preschool children [7].

Numerous studies investigated the long-term consequences of childhood obesity. Childhood obesity has several long-term physical side effects such as atherosclerotic cerebrovascular diseases, colorectal cancers, diabetes, coronary heart diseases, gout, and hip fractures [8]. Additionally, it is associated with enduring mental effects, including reduced years of completed education and lower rates of marriage, among other factors [9].

A substantial body of evidence indicates a clear association between obesity and gynecological disorders in women, as well as sexual disorders in men [10, 11]. Obesity during childhood and adolescence is also linked to early puberty, polycystic ovarian syndrome (PCOS), and menstrual irregularities [12].

Infertility is a failure to conceive after regular unprotected intercourse or attempting pregnancy for a duration exceeding 12 months [13]. Prior research has elucidated an association between obesity and the trajectory of Body Mass Index (BMI) with alterations in sexual function and seminal parameters, including sperm count and concentration [14, 15]. Also, A comprehensive review article has presented compelling evidence indicating an elevated risk of subfertility and aberrant sperm parameters in the individuals afflicted by obesity [16]. A population-based study revealed a twofold increased likelihood of childlessness among males afflicted with obesity between the ages of 17 and 20, in comparison to their counterparts with a normal BMI [17]. Moreover, anthropometric measurements have both direct and indirect influences on the process of sexual partner selection. BMI, physical fitness, and height collectively contribute to the perception of attractiveness [18, 19]. The global concern of male infertility is on the rise, marked by a growing deterioration in the quality of male semen among men residing in Africa, Europe, North America, and Asia [20]. Additionally, there has been a disturbingly rapid rise in the occurrence of female infertility worldwide [21]. We speculated that beyond the currently recognized risk factors for infertility, the disruption might commence at an earlier stage in human life. Hence, there seems to be a possible link between obesity during the formative years and the manifestation of fertility challenges in subsequent stages of life. Despite the existing body of evidence linking childhood obesity to infertility in adulthood, a comprehensive systematic review examining this association has been notably absent. Thus, the primary objective of this study is to investigate the potential correlation between childhood obesity and overweight and the incidence of infertility in later life.

## Methods

This study adhered to the Preferred Reporting Items for Systematic Reviews and Meta-Analyses (PRISMA) guidelines for conducting and reporting systematic reviews [22].

### Search strategy

Systematic literature search was performed from inception up to the end of April 2022 in the international electronic databases including PubMed, Scopus, Web of Science, to search all studies which assessed the association of childhood obesity and infertility in later life. The following terms were chosen and searched with the widest field restriction possible: (“overweight” OR “Obesity” OR “Adiposity” OR “Body mass index” OR “BMI”) AND (“Childhood” OR “children” OR “Adolescent” OR “youth” OR “Adolescence”) AND (“Semen” OR “Sperm” OR “seminal” OR “Fertility” OR “Infertility” OR “reproductive health” OR “sex hormones” OR “Menstruation” OR “Uterine” OR “LH” OR “FSH” OR “Pregnancy” OR “Testosterone” OR “ART” OR “assisted reproductive technology” OR “IVF” OR “in vitro fertilization” OR “adenomyosis”). Google scholar and reference checking was assessed additionally. Furthermore, Google Scholar was manually examined to mitigate the risk of overlooking any additional potentially relevant articles.

### Inclusion criteria

1. Population of interest was children, adolescents, or young adults.
2. Evaluated exposure of the studies was assessing obesity or being overweight during childhood or adolescence or early adulthood.
3. Studies examining various aspects of infertility as an outcome, during adulthood or later stages of life.
4. Study design should be cohort (retrospective or prospective), case-cohort, and nested case-controls.

#### Definition of exposure: obesity

Obesity is characterized by the excessive accumulation of fat within adipose tissue. The diagnosis of obesity is established through the calculation of body mass index (BMI), obtained by dividing an individual’s weight by the square of height. In the case of pediatric populations, obesity is determined by evaluating weight relative to age, acknowledging the evolving body composition during physical growth and development. Numerous nations employ reference charts tailored to their specific populations for weight and height standards. This approach gives rise to varying interpretations of obesity. According to the World Health Organization (WHO), overweight is characterized by BMI-for-age measurements surpassing 1 standard deviation above the WHO Growth Reference median, while obesity is defined by measurements exceeding 2 standard deviations above the same median [23]. Another definition, proposed by the International Obesity Task Force, designates BMI values of 25 for overweight and 30 for obesity at age 18. To accommodate age-specific pediatric groups, LMS multiple-country curves are generated [24].

#### Definition of Outcome: infertility

Infertility was considered in four categories:

- The number of offspring: the number of children.
- Conceiving problems: women who had been attempting to conceive for over 12 months without achieving pregnancy success, or who had sought medical assistance due to difficulties in becoming pregnant.
- Impaired female reproductive system: menstrual disorders, ovulatory disorders.
- Testicular function: sperm quality and quantity.

### Exclusion criteria

1. Papers which did not follow the participants and evaluated outcomes.
2. Participants afflicted with underlying medical conditions known to be associated with infertility.
3. Clinical trials, reviews, animal studies, books, and conference papers.

### Study selection

Records retrieved through search were imported to End Note version 9.3.3. Initially, duplicates were detected and deleted. Two researchers, denoted as (FP) and (ME), conducted the search and initial screening of titles and abstracts based on the previously mentioned inclusion and exclusion criteria. Subsequently, another researcher, identified as (SM), performed a review of the remaining potentially pertinent studies by assessing their full texts. Any disagreements on study selection were discussed and solved by consensus.

### Data extraction

Data were extracted by two authors (FP) and (ME) independently from included studies according to predefined sheets. The extracted data included:

- General characteristics of the studies including first author name, year, country.
- Methodological characteristics of the studies including study design, population of the study, sample size, participant’s sex, duration of follow up, Exposure variable, definition of Obesity.
- outcomes of the studies including infertility or sub-infertility.

### Quality assessment

The quality assessment of the included studies was independently conducted by two researchers, (FP) and (ME), using the Newcastle-Ottawa Quality Assessment Form for cohort, and case-control studies. The Newcastle-Ottawa scale evaluates the methodological quality of the observational studies in eight items within three categories: (1) Selection of participants (2) Comparability of subjects (3) Assessment of outcome/exposure.

## Results

**Fig. 1:**
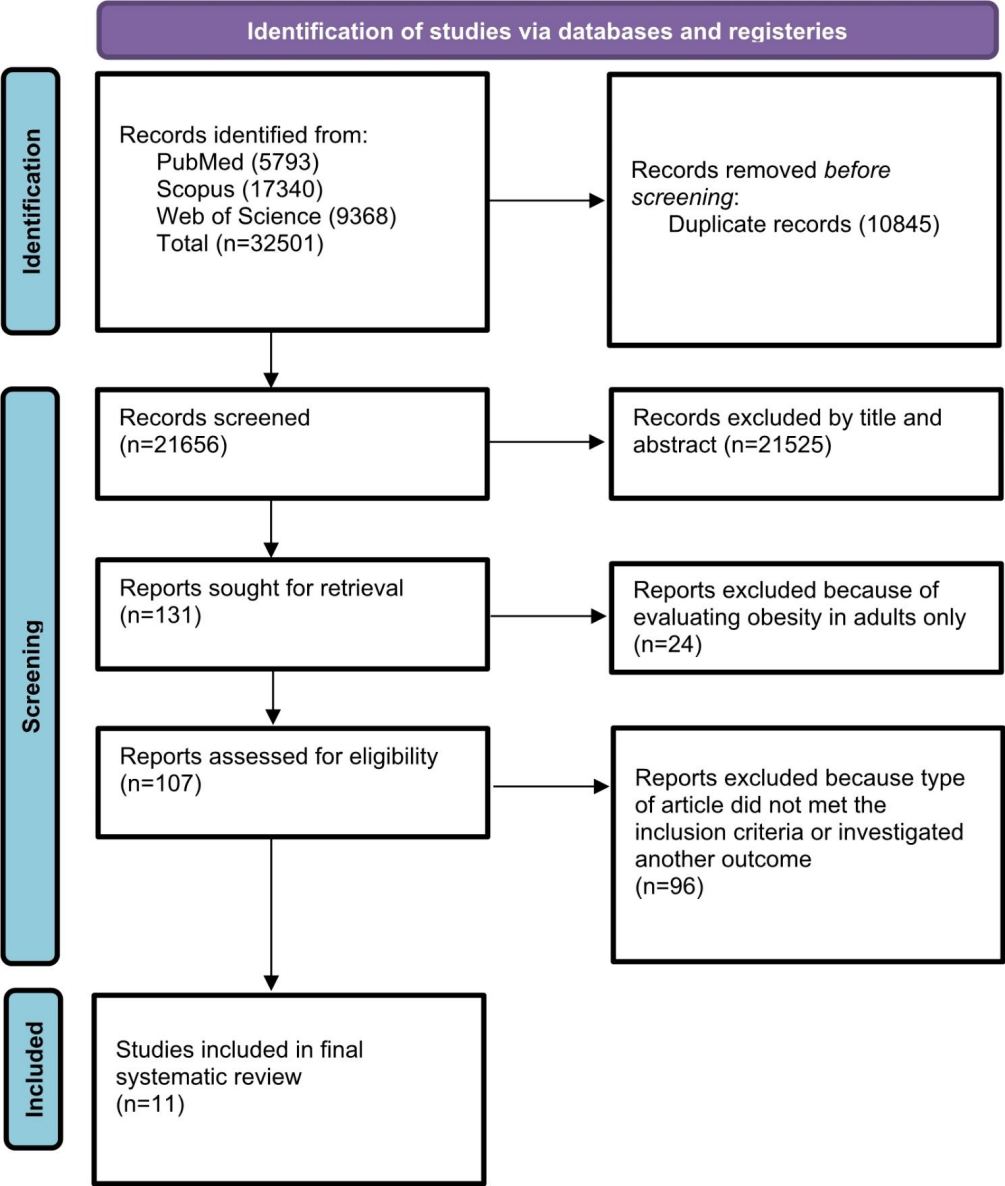
Flow chart for study identification and selection, based on PRISMA 2020 [20]

In total, this systematic review incorporated eleven observational studies. A comprehensive literature search initially identified a pool of 32,501 potentially pertinent articles. After the removal of 10,845 duplicate records and the exclusion of 21,656 articles based on title and abstract screening, 107 studies underwent a through full-text review. Subsequently, a further 96 articles were excluded due to insufficient follow-up data or failure to meet the predefined inclusion criteria. Finally, eleven studies were included in this study. The selection process is visually depicted in Fig. 1.

All the studies were appraised “good” quality using NOS. The details of the quality assessment for the included studies, comprising cohort studies and case-control studies, can be found in the **Supplementary Table.**

. Among the eleven studies reviewed, eight were published within the last 15 years [17, 25–32]. Geographically, six studies were conducted in the United States [25, 27, 28, 30, 31, 33], while the remaining five originated from Australia [29], Sweden [17], United Kingdom [34], Denmark [35], and Finland [26]. To accommodate the broad inclusion criteria employed in the review design, seven of the included articles were prospective cohorts [26–29, 31, 32, 34], three were retrospective cohorts [17, 25, 30], and one was a nested case-control study [33]. Sample sizes exhibited considerable variation, ranging from 193 participants [27] to a substantial 405,427 participants [17]. Total sample size of the included studies is 498,980 participants. Supplementary details regarding the included studies are presented in Table 1. In this study, childhood and early adulthood obesity and overweight are assessed as the exposure variables, with a focus on their impact on four distinct outcome variables: the number of offspring, conceiving problems, impaired female reproductive system, and testicular function.

**Table 1:** General Characteristics of the included studies

References	Country	Population	Study design	sex	Sample size	Duration of follow up (Year)	Exposure variable Definition of Obesity	Outcome investigated	Quality of the studies
**Barclay 2020**	Sweden	17–20 years old Men born in Sweden between 1965–1972	Retrospective Cohort	Male	405,427	44	Overweight BMI : (25-29.99) Obese BMI ≥ 30	Fertility up to age 40 or older	Good
**Frisco 2012**	United States	21–23 Years old females	Retrospective cohort	Female	3,977	20	Overweight BMI : (25-29.99) Obese BMI ≥ 30	Number of children: No children : 0 1 or more live birth: 1	Good
**He 2018**	Australia	Australian school children aged 7–15 years	Prospective cohort	Male and Female	8,498 4,191 Girls 4307 Boys	22.6	Overweight BMI : (25-29.99) Obese BMI ≥ 30, Abdominal obesity was defined as WHtR R0.5	Infertility (was defined as having difficulty conceiving (had tried for ≥ 12 months to become pregnant without succeeding) or having seen a doctor because of trouble becoming pregnant)	Good
**Jacobs 2016**	United States	Children aged 5 to 14 years old	Prospective cohort	Female	5,824	33	85 ≤ BMI percentile ≤ 94 : Overweight BMI Percentile ≥ 95 : Obese Subscapular and triceps skinfold thicknesses percentile: High : 85 ≤ Percentile ≤ 94 Very high: 95 ≥ Percentile	Fertility difficulties: The women who answered Yes two any of the 3 question below were considered infertility difficulty group: 1.Taking any fertility drugs 2.Trying to become pregnant and were unable to 3.visiting doctor for becoming pregnant	Good
**Jokela 2008**	United States	17–24 years old young adults	Prospective cohort	Male and Female	12,073 5982 women 6091 men	23	Overweight BMI : (25-29.99) Obese BMI ≥ 30	Number of children desired	Good
**Kahn 2019**	United States	Children from health and development cohort until 44 Years of old	Prospective cohort	Male	193	44	Overweight BMI : (25-29.99) Obese BMI ≥ 30	Sperm concentration, motility, and morphology	Good
**Lake 1997**	United Kingdom	Children born on 3rd to 9th of March 1958	Prospective cohort	Female	5,799	26	Age specific BMI cut-off	Menstural problems, Subfertility, HTN Subfertility defined as time taken to conceive from cessation of contraception	Good
**Laru 2021**	Finland	Women born in 1966	Prospective cohort	Female	4,382	50	85 ≤ BMI percentile ≤ 94 : Overweight BMI Percentile ≥ 95 : Obese	Impaired reproductive function defined as decreased fecundability, need for infertility assessment and treatment by 46 years of age. Childlessness and number of children also extracted.	Good
**Polotsky 2010**	United States	Women aged between 42–52	Retrospective cohort	Female	3,302	NR	Overweight BMI : (25-29.99) Obese BMI ≥ 30	Nulliparity and nulligravidity	Good
**Ramlau-Hansen 2010**	Denmark	5–8 Years	Retrospective Cohort	Males	260	22	High prepubertal BMI (16.21–22.58 kg/m2)	Sperm Concentration, Sperm volume, Sperm total count, percent normal morphology sperm, percent motile sperm, Testosterone, Estradio, FSH, LH, SHBG, Inhibin B.	Good
**Rich-Edwards 1994**	United States	Females registered nurses 25 to 42 years old	Nested case-control study	Female	2,527 Case 46,718 Control	NR	BMI categories	Infertility due to ovulatory disorder	Good

BMI: Body Mass Index, FSH: Follicle Stimulating Hormone, LH: Luteinizing Hormone, SHBG: Sex-Hormone Binding Globulin

### Number of offspring

The number of children was the primary focus in five studies [17, 25, 26, 28, 30].

Frisco et al. conducted their study by analyzing data from the female sample of the U.S. National Longitudinal Study of Youth. They recruited two distinct groups of females, Cohort 1, and Cohort 2, who had entered early adulthood (aged 21–23) during two different time periods in the 1980s [30]. In Cohort 2, the prevalence of overweight and obesity among women was higher, and though the difference was modest, it was deemed statistically significant. Frisco et al.‘s study outcomes indicated that within Cohort 1, for each increase in BMI during early adulthood, there was a 5.9% reduction in the odds of experiencing a first birth between the ages of 21–23 and 41–43. Jokela et al. conducted a longitudinal study that tracked American young adults aged 17–24 years in 1981 through the year 2004. Their research findings revealed that obese women were less likely to have any children [28]. Additionally, the study by Jokela et al. observed that obese men were also less likely to have their first and second child. However, there was no significant difference in the probability of having a third or fourth child when compared to normal-weight men. Furthermore, the study conducted by Jokela et al. included adjustments for variations in marital status over the life course. These adjustments revealed that, among obese women, the probability of having the first and third child remained lower. However, it’s noteworthy that the association between obesity in women and having a first or fourth child was not statistically significant. In contrast, the study did not find any significant impact on the association between men’s fertility and obesity after adjusting for marital status. In the study by Polotsky et al., they reported a notable trend of a progressive increase in lifetime nulliparity across the high school BMI categories. This suggests that higher BMI levels during high school were associated with a greater likelihood of individuals not having children during their lifetime [25]; Furthermore, Polotsky et al.‘s study revealed that adolescent obesity was linked to both lifetime nulliparity (the state of never having given birth) and nulligravidity (the absence of pregnancy) even after comprehensive adjustments were made. These adjustments included accounting for BMI in adulthood, a history of nongestational amenorrhea (lack of menstruation unrelated to pregnancy), marital status, ethnicity, study site, and various socioeconomic status measures. This underscores the robustness of the association between adolescent obesity and fertility outcomes. Barclay et al. conducted a study utilizing data from Swedish population and military conscription registers. Their primary aim was to investigate the relationship between health in early adulthood and male infertility [17]. Their findings led to the conclusion that the probability of childlessness was nearly twice as high in men who were obese during the ages of 17–20 when compared to individuals with a normal BMI in the same age group. Fecundability was evaluated based on the number of children women had by age 50 among those who had been enrolled in Laru et al.‘s study during childhood [26]; Obesity during the age intervals of 7–10 [adjusted odds ratio (OR) = 2.05 (1.26–3.35)] and 11–15 [adjusted OR = 2.04 (1.21–3.44)] was associated with fewer children born to the participants. Furthermore, even after excluding women with polycystic ovary syndrome (PCOS), there was a heightened risk of childlessness among participants who were either overweight or obese during the age range of 11–15 [adjusted OR = 1.56 (1.06–2.27), adjusted OR = 1.77 (1.02–3.07)].

### Conceiving problems

Two of the studies included in the analysis assessed self-reported fertility difficulties by utilizing completed questionnaires as their primary data collection method. In the study conducted by He et al., women who had been attempting to conceive for over 12 months without achieving pregnancy success, or those who had sought medical assistance due to difficulties in becoming pregnant, were categorized as experiencing infertility [29]. Their findings indicated that childhood obesity before the age of 12 could substantially elevate the risk of female infertility later in life, with an adjusted relative risk (aRR) of 2.94 and a 95% confidence interval (CI) of 1.48–5.84. Jacobs et al. proposed a connection between childhood adiposity (excess body fat) and self-reported fertility difficulties [31]; The study found that women who were obese between the ages of 9 and 12 were more likely to experience fertility troubles, with an adjusted relative risk (aRR) of 1.82 and a 95% confidence interval (CI) of 1.17–2.82.

### Impaired female reproductive system

Two of the reviewed publications undertook investigations into female reproductive disorders and gestational problems as potential outcomes associated with obesity during adolescence and early adulthood [33, 34]. Lake et al.‘s study unveiled that obesity at both age 7 (odds ratio, OR = 1.59) and age 23 (OR = 1.75) may independently raise the risk of experiencing menstrual problems by the age of 33. Furthermore, following adjustments for confounding factors, it was observed that the risk of hypertension during pregnancy significantly increased among participants who were obese at age 23, with an odds ratio (OR) of 2.37 [34]. Rich-Edwards et al. conducted an examination of the association between BMI at age 18 and the occurrence of subsequent infertility attributed to ovulatory disorders [33]; In this nested case-control study, obesity at the age of 18 emerged as a notable risk factor for subsequent ovulatory infertility. The study’s findings revealed multivariable relative risks for infertility, which increased progressively with higher BMI categories (BMI < 16 RR = 1.2, BMI 26 to 27.9 RR = 1.7 and BMI ~ 32 RR = 2.7). These relative risks indicate an increasing likelihood of infertility with higher BMI values, particularly highlighting the significant impact of obesity at age 18 on the risk of ovulatory infertility in later life.

### Testicular function

In the study conducted by Kahn et al., testicular function was assessed. Their findings provided support for the notion that obesity during early adulthood plays a role in impaired testicular function [27]. In this specific study, it was found that obesity in men during their 20s was linked to an elevated risk of low motility of sperm [odds ratio, OR = 2.4 (1.3–4.4)]. Additionally, there was an even higher risk of poor sperm morphology in semen analysis [OR = 1.9 (0.94–3.8)] associated with obesity in this age group. These findings underscore the potential impact of obesity on male reproductive health parameters, particularly in relation to sperm motility and morphology.

The study by Ramlau-Hansen et al., was conducted on 347 Danish young men, aimed to investigate the associations between birth weight, prepubertal (around 5 to 8 years of age) BMI, and adult BMI with semen quality and sex hormone levels. The research encompassed semen analysis and blood sample collection as part of its data collection process. The findings did not reveal any significant correlations between birth weight and either semen quality or hormonal levels. Prepubertal BMI did not show significant patterns associated with semen quality; however, it did demonstrate a connection with decreased concentrations of SHBG (Sex Hormone-Binding Globulin). [Adjusted mean for SHBG (nmol/L): Low BMI: 30.8 (26.7, 35.4), Medium BMI: 28.8 (25.0, 33.0), High BMI: 26.2 (22.6, 30.1), *p* value: 0.005] [35].

The main findings and adjustments of the included studies are listed in Table 2.

**Table 2:** Main Findings of included studies and adjustments

	Study	Main Findings	Adjustment
**1**	**Barclay 2020**	Normal BMI = Reference group (20 < BMI < 21.99) **Obese (BMI ≥ 30) final parity** = **ß :-0.682** (SE = 0.058) (CI: -0.796 to -0.568)	Birth cohort, Birth year, Convenience age, Age at time of the conscription, Completed sibling group size, education; attainment education, Cumulative income
**2**	**Frisco 2012**	Early adult BMI is negatively and significantly related to ever having a birth among Cohort 1 population. Each increase in BMI = ↓ Odds of first birth by 5.9% (1-exp (-0.061)). Early adult BMI is not significantly associated with first birth among Cohort 2 population.	Race/ethnicity, women’s educational background and their mothers’ years of education, women’s age of menarche, fertility expectations
**3**	**He 2018**	Obese group compared to normal BMI group (Ref): **Tried for > 12 month to become pregnant**: RR:3.899 (CI:1.95–7.77)	Childhood age, follow-up length, highest parental education, and marital status
		Obese group compared to normal BMI group (Ref): **Seen a doctor due to trouble become pregnant** : RR :2.36 (CI:0.95–5.85)	
		Obese group compared to normal BMI group (Ref): **Infertility**: RR : 2.94 (CI:1.48–5.84)	
**4**	**Jacobs 2016**	Child **BMI (< 9 Years)** Obese compared to normal BMI (Ref): **Infertility (Tried Unable)** : RR = 1.76(CI:1.04–2.97)	Education level, race, tobacco use history, current BMI and adult income
		Early teen **BMI (9–12 Years)** Obese compared to normal BMI (Ref): **Infertility (Tried Unable)** : RR = 1.94(CI:1.22–3.08)	
		Late teen **BMI (13–18 Years)** Obese compared to normal BMI (Ref): **Infertility (Tried Unable)** : RR = 1.44(CI:1.03–2.03)	
**5**	**Joleka 2008**	Obese women compared to normal weight (Ref) for : **First child** = RR :0.87(CI:0.79–0.96) **Second child** = RR:0.90(0.76–1.05) **Third child** = RR: 0.74(0.58–0.94) **Forth child** = RR : 0.65(0.39–1.08)	Race/ethnicity, subsample membership, and urban residence
		Obese men compared to normal weight (Ref) for : **First child** = RR :0.78(CI:0.69–0.88) **Second child** = RR:0.78(0.67–0.91) **Third child** = RR: 1(0.79–1.26) **Forth child** = RR : 0.91(0.77–1.07)	
**6**	**Kahn 2019**	**BMI at 4 years** ≥ 85 percentile compared to < 85 percentile: **Sperm < 15 million/ml**: OR:1.7(CI:0.55-5) **Progressive motility < 32%**: OR:1.4(0.67–2.8) **Sperm morphology < 4% normal**: OR:1.4(0.6–3.1)	Birth weight, gestational age, abstinence time, and age at examination
		**BMI at 20s years** ≥ 25 kg/m^2^ compared to < 25 kg/m^2^: **Sperm < 15 million/ml**: OR:1.4(CI:0.54–3.6) **Progressive motility < 32%**: OR:2.4(1.3–4.4) **Sperm morphology < 4% normal**: OR:1.9(0.94–3.8)	
**7**	**Lake 1997**	Obese women at 23 year were less likely to conceive within 12 months of unprotected intercourse after adjustment for confounders **(RR = 0.69)**	Social class at birth, 7 and 23 y; smoking at 23 y; parental education; parity; menstrual problems at 16 y; and age of menarche
**8**	**Laru 2021**	**Obesity at ages 7–10 years: Decreased fecundability at age 31** : RR = 2.05(CI:1.26–3.35), **Childlessness at age 51 years** : RR = 1.15(CI:0.68–1.94)	Marital status at ages 31 and 46 years, and smoking and education level at age 31 years
		**Obesity at ages 11–15 years: Decreased fecundability at age 31** : RR = 2.04(CI:1.21–3.44), **Childlessness at age 51 years** : RR = 1.97(CI:1.19–3.27)	
**9**	**Polotsky 2010**	Obese group compared to normal BMI group: **Nulliparity: OR:2.84(1.59–5.10)**	Adult BMI, history of nongestational amenorrhea, marital status, ethnicity, study site, and measures of socioeconomic status
		Obese group compared to normal BMI group: **Nulligravidity: OR:3.93(2.12–7.26)**	
**10**	**Ramlau-Hansen 2010**	BMI tertile: Low BMI (12.8522–15.2348) Medium BMI (15.2355–16.1941) High BMI (16.2174–22.5851) Childhood (ages 5–8 years) BMI were not significantly associated with **semen quality** at age 18–21. **Semen quality: Sperm concentration (millions/mL), Semen volume (mL), Sperm total count (millions), Percent normal morphology sperm, Percent motile sperm** Men with the 33% highest childhood BMI had 15% lower sex hormone binding globulin **([adjusted mean for SHBG (nmol/L): Low BMI: 30.8 (26.7, 35.4), Medium BMI: 28.8 (25.0, 33.0), High BMI: 26.2 (22.6, 30.1), p value: 0.005])**	Season (summer/winter), history of diseases of the reproductive organs (cryptorchidism, hypospadias, varicocele, hydrocele, orchitis, and chlamydia combined into one variable, present or not present), smoking and maternal smoking during pregnancy. The semen outcome variables were additionally adjusted for abstinence time and spillage during collection of the sample. The results on motility were also adjusted for time from ejaculation to analysis. The blood sample outcome variables were, in addition to season, diseases of the reproductive organs, and own and maternal smoking, also adjusted for time of day of blood sampling.
**11**	**Rich-Edwards 1993**	Multi variate RR for obese group compared to controls **Ovulatory infertility** = RR:2.7(CI:2-3.7)	Age, year of birth, ethnicity, frequency of physical activity at ages18-22, smoking status at ages 15–19, alcohol use at ages 18–22, DM and use of OCP before age 22

BMI:Body Mass Index,CI:Confidential Interval, RR: Relative Risk, Ref:Referrence,Y:Year,DM:Diabetes Mellitus,OCP:Oral Contra Ceptive.

## Discussion

The findings from the current review provide substantial evidence supporting a positive association between obesity during childhood, adolescence, and potentially even early adulthood, and the occurrence of fertility troubles in both men and women. Despite the inherent heterogeneity among the eleven studies included in this review, a consistent positive relationship emerged across all of them, except one controversial study [35]. This relationship pertains to being overweight or obese during early life and experiencing challenges in fecundity. However, it’s important to note that half of the included articles primarily focused on the number of children, which cannot be equated directly with fertility problems. Nonetheless, collectively, the evidence strongly suggests that early-life obesity is associated with reproductive difficulties.

Among the studies included in this analysis that utilized the number of children as an indicator of fertility, four of them collected anthropometric data from adolescents or young adults, often students attending high school. Only one prospective study enrolled participants aged between 7 and 15 years old. It is plausible to suggest that these variations in the number of children among individuals with different BMI levels, particularly in early adolescence or adulthood, may be influenced by factors related to sexual and romantic partnerships. As expected, it is possible that obese adolescents may have fewer opportunities to develop intimate romantic relationships, which could contribute to differences in fertility outcomes [36].

In two of the studies included in our analysis, several thousand females, aged between 7 and 12 at the time of recruitment, were enrolled. These individuals were followed up over the years, and their identification as potential cases of fertility difficulties was based on criteria such as seeking medical assistance due to conceiving difficulties, using fertility drugs, or undergoing medical procedures related to fertility concerns [29, 31]. In one of the studies, crucial confounding factors, including education levels, race, tobacco use history, adulthood BMI, and income, were meticulously adjusted for. This rigorous approach ensures that the impact of early-life obesity on fertility outcomes is assessed while controlling for potential influences from these important variables [31]. In the other study, the researchers went a step further and adjusted for marital status, acknowledging its potential influence on fertility outcomes. This comprehensive adjustment helps to isolate the specific impact of early-life obesity on fertility while considering the potential mediating effect of marital status [29]. The inclusion of adulthood BMI as an adjustment factor in the study by He et al. adds significant value to their conclusions. This adjustment is particularly important because there is already substantial evidence highlighting the influence of adulthood obesity on infertility. By accounting for adulthood BMI, the study can more precisely isolate the unique impact of early-life obesity on fertility outcomes, providing valuable insights into this specific aspect of the relationship between obesity and infertility [29, 37]. In the study by Lake et al., the researchers took into consideration the presence of hypertension during pregnancy as a potential risk factor that could complicate not only pregnancy but also the childbirth process and the postpartum period (puerperium). This comprehensive approach underscores the importance of understanding how obesity during early life may contribute to various pregnancy-related complications, including hypertension [34]. It is indeed worth mentioning that pre-pregnancy obesity represents a significant risk factor for gestational hypertension. It is imperative that obese women receive appropriate counseling and are made aware of their elevated risk for this medical complication during pregnancy. Additionally, given the potential threat of congenital abnormalities to the fetus, it underscores the importance of conducting thorough screening and monitoring for any potential issues to ensure the well-being of both the mother and the unborn child [38].

infertility can have a profound impact on an individual’s mental and emotional well-being, particularly in women. It can lead to significant psychological distress, stress, anxiety, and even depression. The journey to conceive can be emotionally taxing, and the inability to achieve pregnancy as desired can be a source of frustration and sadness [39, 40]. Obesity constitutes a substantial and adverse health condition with wide-ranging implications for reproductive health. In females, obesity can give rise to a plethora of issues that profoundly affect the reproductive process, encompassing challenges related to oocyte quality, hormonal imbalances, metabolic disruptions, and endometrial dysfunction. This multifaceted impact on reproductive health is of paramount significance. Notably, the prevalence of infertility in women has risen to a concerning range of 10–17% [41, 42]. Polycystic Ovary Syndrome (PCOS) stands as the primary contributor to approximately 90% of cases of anovulatory infertility, where ovulation does not occur as expected. Furthermore, it is essential to recognize that women affected by both PCOS, and obesity may experience reduced chances of conception and diminished success rates when utilizing assisted reproductive techniques. This emphasizes the intricate relationship between PCOS, obesity, and fertility, underscoring the need for tailored approaches to address the unique challenges faced by individuals in this group [43, 44].

In overweight women, the likelihood of achieving pregnancy decreases by a minimum of 8%, while in obese women, this reduction is even more substantial, at 18%. This highlights the significant impact of excess weight on fertility and the importance of weight management in addressing fertility challenges [45, 46]. Insulin resistance serves as the primary pathological mechanism that establishes the crucial link between obesity and anovulation, ultimately leading to infertility [47, 48]. Insulin exerts inhibitory effects on hepatic sex-hormone binding globulin (SHBG) and insulin-like growth factor binding protein (IGFBP) synthesis, resulting in elevated levels of free sex steroid hormones. Furthermore, insulin contributes to heightened production of insulin-like growth factor (IGF) primarily from the liver. This increased IGF production stimulates ovarian steroidogenesis. Consequently, elevated gonadal steroid levels disrupt normal gonadotrophin secretion, adversely affecting both folliculogenesis and ovulation processes. These complex interactions highlight the intricate role of insulin in modulating reproductive function and fertility [49, 50]. Another concern in obese women is the role of increased serum leptin levels. Elevated leptin can disrupt the regulation of gonadotrophin-releasing hormone (GnRH) and lead to alterations in ovarian steroidogenesis. This dysregulation can further contribute to fertility challenges in individuals dealing with obesity, underlining the multifaceted nature of the relationship between adipose tissue, hormones, and reproductive function [51].

In the context of male infertility, obesity can exert its impact through various mechanisms, including hormonal imbalances, disrupted spermatogenesis (the process of sperm production), reduced semen quality, sperm DNA fragmentation, and the potential onset of erectile dysfunction [52]. Obesity-induced systemic inflammation is closely associated with male infertility, with hypogonadism being a significant comorbidity. This inflammatory response involves a range of immune mediators, including interleukin (IL)-1β, IL6, IL8, IL12, tumor necrosis factor-alpha (TNFα), interferon-gamma (IFNγ), transforming growth factor-beta (TGFβ), macrophage inflammatory protein (MIP-1), and monocyte chemotactic protein (MCP-1) [53]. Additionally, leptin has the potential to induce macrophage infiltration, which can in turn trigger an inflammatory response. This cascade of events may lead to hypothalamic inflammation. The alteration of hypothalamic-releasing hormones subsequently dysregulates the hypothalamic-pituitary-gonadal (HPG) axis, a pivotal hormonal system involved in reproductive function [54]. As expected, Ramalau-Hansen et al. demonstrated association between childhood BMI and impaired reproductive hormones [35]. However, the same association was not observed between pre-pubertal BMI and semen quality. As the authors have explained, there were some limitations in their study which might influence results and cause bias. For instance, due to few obese or underweight participants, instead of using accepted WHO limits to set exposure groups, they categorized BMI using tertiles. Also, the participation rate was 48.5% that is not high enough to eliminate the risk of selection bias. In addition, subjects were too young (18–21 years old) and mostly did not have reproductive experience. Other considerable factor is that semen data are disposed to measurement errors and biologic variation.

Lifestyle choices, exercise routines, and dietary patterns have a direct influence on obesity. In a study conducted by Chavarro et al., it was observed that a diet rich in nutrients, effective weight control, and regular physical activity were associated with a significant 69% reduction in the risk of ovulatory disorders [55]. When it comes to the effects of exercise, a substantial body of research has shown that while exercise provides numerous health benefits for most women, it can also have adverse effects on the female reproductive system. In situations where the body is pushed into a catabolic state due to excessive exercise, it may prioritize overall health by suppressing the reproductive system. This can lead to menstrual abnormalities, ovulatory dysregulation, and potential issues with the quality of oocytes. However, it’s important to note that making definitive claims about the association between exercise and male infertility requires well-designed studies, which are currently lacking. Nevertheless, it’s worth acknowledging that excessive physical activity may have detrimental effects on male fertility, even though the research in this area is not as extensive as it is for female fertility [56]. Regarding dietary patterns, a systematic review conducted by Salas-Huetos et al. that analyzed 35 studies revealed several interesting findings. They reported that diets incorporating fish, shellfish, seafood, poultry, cereals, vegetables, fruits, low-fat dairy, and skimmed milk were positively associated with various sperm quality parameters. Conversely, diets rich in processed meat, soy foods, potatoes, full-fat dairy, total dairy products, cheese, coffee, alcohol, sugar-sweetened beverages, and sweets were associated with lower semen quality in some of the studies they included. These findings emphasize the potential impact of dietary choices on male reproductive health, highlighting the importance of a balanced and nutrient-rich diet for maintaining optimal sperm quality [57].

Almost all the evidence in our study has shown that being obese in a period of time during early age might increase the risk of infertility in adulthood. Infertility not only affects individuals’ physical and mental health, but also can bring a huge financial burden looking for its treatment. On the other hand, obesity is a condition that can be treated easier than infertility. These results confirm the importance of active prevention and treatment of obesity during childhood, adolescence, and early adulthood. Findings of this study is a warning for health authorities to take action and make fundamental policies in different levels such as public health, universities, schools, even in family by educating parents.

It’s noteworthy that all the studies included in this review have been assessed positively in terms of their quality based on legitimate quality assessment scales. Secondly, the duration of the follow-up in most of the studies was more than 20 years. However, it’s important to acknowledge that the heterogeneity of the outcomes investigated across these studies may pose a limitation to meta-analyze the data. Additionally, the relatively small number of articles included in this review is another limitation.

To further strengthen and corroborate the findings of this review, there is a need for more cohort studies with long follow-up durations in this field. This would enhance the robustness and comprehensiveness of the evidence base and provide a more comprehensive understanding of the relationship between obesity in early life and its impact on reproductive health.

## Conclusion

The published evidence suggests a positive association between early-life obesity and reduction of fecundity and even maybe infertility in later life. Unveiling the impact of obesity in childhood and adolescence on a crucial consequence like fertility might potentially make children and adolescents and their parents more motivated to always keep an eye on their weight.

### Electronic supplementary material

Below is the link to the electronic supplementary material.

Supplementary Material 1

## Data Availability

The datasets generated during the current study are not publicly available due to ethical concerns but are available from the corresponding author on reasonable request.

## Abbreviations

ART: Assisted Reproductive Technology
BMI: Body Mass Index
DNA: Deoxyribonucleic acid
FSH: Follicle Stimulating Hormone
GnRH: Gonadotrophin-Releasing Hormone
HPG: Hypothalamic- Pituitary-Gonadal
HSE: Hanieh-Sadat Ejtahed
IFN: Interferon
IGF: Insulin Like Growth Factor
IGFBP: Insulin-Like Growth Factor Binding Protein
IL: Interleukin
IVF: In Vitro Fertilization
LH: Luteinizing Hormone
MCP: Monocyte Chemotactic Protein
MIP: Macrophage Inflammatory Protein
OR: Odds Ratio
PCOS: Poly Cystic Ovarian Syndrome
PRISMA: Preferred Reporting Items for Systematic reviews and Meta-Analyses
RR: Relative Risk
SHBG: Sex-Hormone Binding Globulin
TNF: Tumor Necrosis Factor
US: United States
