# Supplementary material for: Association between childhood obesity and infertility in later life: a systematic review of cohort studies
Source: BMC Endocr Disord. 2023 Oct 24;23:235. doi: 10.1186/s12902-023-01490-4 (PMC10594820; doi:10.1186/s12902-023-01490-4)
Supplement: Supplementary file 1 — Supplementary Material 1 [file 12902_2023_1490_MOESM1_ESM.docx]

**Supplementary Table 1**. Quality Assessment of included studies according to Newcastle Ottawa Quality Scale.

|  | **Selection** | | | | **Comparability** | **Outcome** | | | **Overall Quality** |
| --- | --- | --- | --- | --- | --- | --- | --- | --- | --- |
|  | **Representive** | **Non-exposed** | **Exposure** | **Outcome of interest** | **Comparability** | **Assessment** | **Duration of F/U** | **Adequecy of F/U** |  |
| **Barclay 2020** |  | * | * | * | ** | * | * | * | Good |
| **Frisco 2012** |  | * | * | * | ** | * | * | * | Good |
| **He 2018** | * | * | * | * | ** | * | * | * | Good |
| **Jacobs 2016** | * | * | * | * | ** | * | * | * | Good |
| **Jokela 2008** | * | * | * | * | ** | * | * | * | Good |
| **Kahn 2019** |  | * | * | * | ** | * | * | * | Good |
| **Lake 1997** | * | * | * | * | ** | * | * | * | Good |
| **Laru 2021** | * | * | * | * | ** | * | * | * | Good |
| **Polotsky 2010** | * | * | * | * | ** | * | * | * | Good |
| **Ramlau-Hansen 2010** | * | * | * | * | ** | * | * |  | Good |
| **Rich-Edwards 1994** | * | * | * | * | ** | * | * | * | Good |

Good quality: 3 or 4 stars in selection domain AND 1 or 2 stars in comparability domain AND 2
or 3 stars in outcome/exposure domain
Fair quality: 2 stars in selection domain AND 1 or 2 stars in comparability domain AND 2 or 3
stars in outcome/exposure domain
Poor quality: 0 or 1 star in selection domain OR 0 stars in comparability domain OR 0 or 1 stars
in outcome/exposure domain
